# Roles of contrast-enhanced ultrasonography in identifying volume change of benign thyroid nodule and optical time of secondary radiofrequency ablation

**DOI:** 10.1186/s12880-020-00476-1

**Published:** 2020-07-14

**Authors:** Ziyu Jiao, Yukun Luo, Qing Song, Lin Yan, Yaqiong Zhu, Fang Xie

**Affiliations:** 1 Department of Ultrasound, First Medical Center, Chinese PLA General Hospital, 28 Fuxing Road, Haidian District, Beijing, 100856 China; 2 Department of Ultrasound, Seventh Medical Center, Chinese PLA General Hospital, 5 Nan Mencang Road, Dongcheng District, Beijing, 100700 China

**Keywords:** Benign thyroid nodule, Contrast-enhanced ultrasonography, Secondary radiofrequency ablation

## Abstract

**Background:**

Ultrasonography-guided radiofrequency ablation (RFA) was was proved to be an effective and safe treatment with few complications for benign thyroid nodule. In cases of incompletely treated nodule margin, secondary RFAs are necessary. The present study was designed to analyze the dynamic change of nodular volume of benign thyroid tumors accessed using contrast-enhanced ultrasonography (CEUS) after RFA, and hopefully to offer evidence for time decision of secondary RFA.

**Methods:**

A total of 105 patients who received ultrasonography-guided RFA in the Department of Ultrasound, Chinese PLA General Hospital between April 2014 and August 2018 for benign thyroid nodule were enrolled in this retrospective study. Vt increase (regrowth) and vital volume (Vv) of thyroid nodule were followed up at 0, 1, 3, 6, 12, 24 and 36 months after RFA.

**Results:**

A total of 105 nodules of 105 patients were enrolled in the present study, with a mean age of 46.70 ± 13.05 years, and 87 of them (82.9%) were female. The median follow up time was 25.1 months (12 months to 36 months). During the follow up, regrowth occurred in 43 cases, 95.35% of nodular regrowth occurred in 12 months after RFA, and the rate showed substantial consistency with that on the 36th month postoperatively (Kappa = 0.656).

**Conclusions:**

CEUS was an effective and safe tool to monitor volume change of benign thyroid nodules after RFA. The majority cases of regrowth occurred in 12 months after RFA, thus, the 12th month after RFA might be the optimal time for volume assessment to make the decision of secondary RFAs.

## Background

In the past few years, ultrasonography-guided radiofrequency ablation (RFA) was proved to be an effective and safe treatment for both benign and malignant thyroid tumor, with few complications using trans-isthmic approach and moving-shot technique [1–4]. According to previous studies, a 50–80% volume reduction of thyroid nodule after 6 months [5], a 79–90% volume reduction after 2 years [6], and a 93% volume reduction after 4 years [7] could be observed postoperatively, with a longest and largest follow-up of 5 years reported [8].

Although satisfying volume reduction rate and symptomatic and/or cosmetic improvement could be achieved using RFA, previous studies had reported a recurrence rates of 5.6% for benign tumors, and all recurrent cases showed regrowth of incompletely treated nodule margin [7], which was considered as a reason of nodule recurrence after RFA [9].

Since a complete nodule margin is extremely important for prevention of nodular recurrence [10], in cases with incompletely treated nodule margin, a secondary RFA is should be necessary and beneficial. A secondary RFA was usually performed when symptom resolution was not achieved, residual tissue regrowth was noted or increasing vascularity was obviously observed. However, no consensus on the optimal itme to perform the secondary RFA was achieved due to lack of evidence. Thus, the current study was designed to analyze the dynamic change of nodular volume of benign thyroid tumors accessed using contrast-enhanced ultrasonography (CEUS) after RFA, and hopefully to offer evidence for time decision of secondary RFA.

## Methods

### Patients

A total of 105 patients who received ultrasonography-guided RFA in the Department of Ultrasound, Chinese PLA General Hospital between April 2014 and August 2018 for benign thyroid nodule were enrolled in this retrospective study. Inclusion criteria were as followed: 1) nodules were confirmed to be benign by at least two cases of definite needle aspiration cytology/biopsy [11]; 2) serum calcitonin levels were tested to be normal. Exclusion criteria were as followed: 1) severe coagulation dysfunction; 2) Left ventricular ejection fraction < 50%, alanine aminotransferase > 3 times upper limit of normal reference, and creatinine clearance rate < 30 ml/min; and 3) severe neck skin infection; 4) nodules diagnosed as warm nodules in nuclide imaging; 5) multi-nodules in the same patient. The current study was approved by the Ethics Committee of Chinese PLA General Hospital. All participants provided written informed consents.

### Procedures

All the procedures, including ultrasonography-guided cytology/biopsy and ultrasonography guided radiofrequency ablation were performed by the same radiologist who have performed with more than 20 years’ experience of thyroid ultrasonography and intervention ultrasound, and ultrasonography-guided cytology/biopsy were performed in accordance of previously published technical concensus and recommendations [12–14]. Similar procedure were also reported in our previous studies [2, 15].

Preoperative conventional ultrasonography and postoperative CEUS were performed using a high-frequency linear probe (L12–4, Mindray M9, China). Ultrasonography guidance for RFA were performed using a high-frequency linear probe (6 L3, Siemens 512, Germany), and RFA were performed using a internally cooled bipolar electrode with a 9 mm or 15 mm active component (100-T09, CelonProSurge and Celon LabPOWER, Olympus Surgical Technologies Europe, Hamburg, Germany). For CEUS, Sulphur hexafluoride with a phospholipid shell (SonoVue®, Bracco, Milan, Italy) was diluted using normal saline (0.9%, 5 ml), and a bolus injection (2.4 ml) followed by a flush of normal saline (0.9%, 5 ml) were used for administration.

Three orthogonal diameters were measured between the outer margins of the nodules. A length value was measured on a sagittal image, whereas the width and height were measured on an axial image [16]. The volume was calculated according to the following formula: V = Πabc/6 (V: volume, a: the largest diameter, b and c: the other two perpendicular diameters) [17–19].

During RFA, patients had their necks fully extended in a supine position. Trans-isthmic approach and moving-shot technique were applied after local anaesthesia with 1% lidocaine. For thyroid nodule with cystic portion, fluid was aspirated as much as possible prior to RFA, which was performed for remaining solid portion. CEUS was conducted immediately after RFA to evaluate whether complete ablation of the nodule was achieved, which appeared as non-enhanced area. Additional RFA was conducted if residual area was observed. Vital signs of patients were observed for 1–2 h after RFA, and the occurrence of complications, such as voice change and local haematoma, were recorded [20].

Volume change of thyroid nodule was followed up at 0, 1, 3, 6, 12, 24 and 36 months after RFA. Three types of nodule volumes were defined based on the following criteria: 1) Vt: total volume of the nodule; 2) Va: volume of ablated area (non-enhanced area); and 3) Vv: volume of unablated area (Vt - Va). The primary endpoint was sign of regrowth by evaluating total volume (Vt), ablated volume (Va) or vital volume (Vv) of treated nodule, which was defined as an more than 50% increase of Vt or Vv compared to their previous smallest volume [7, 11, 21].

### Statistics

Continuous variables with normal distribution were described using mean and standard deviation, and those with skewed distribution were described using median and interquartile range. Categorical variables were described using number and percentage. Mcnemar’s test was used for compare of accumulated rate of regrowth in different time points, and kappa coefficient was used for the evaluation of their agreement. Statistic Package for Social Science version 18.0 software (SPSS Inc., Chicago, USA) was applied to conduct statistical analysis, and difference was considered as statistically significant when *p* < 0.05.

## Results

### The general characteristics of nodules

**Fig. 1:**
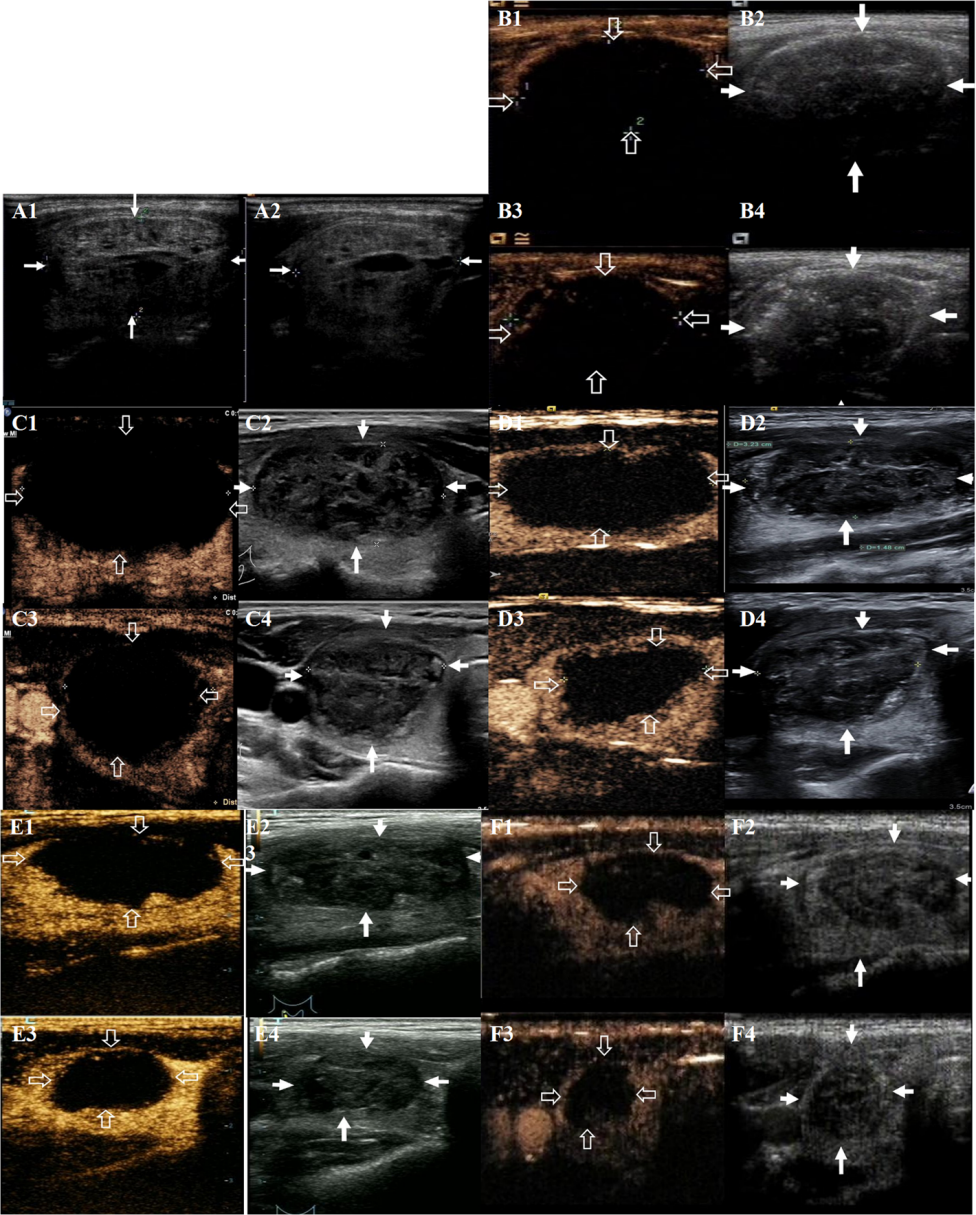
Preoperative and postoperative ultrasonography of one patient. A: preoperative ultrasonography (A1: longitudinal section of two-dimensional ultrasonography; A2: transverse section of two-dimensional ultrasonography); B: postoperative ultrasonography at 0 months (A1: longitudinal section of contrast-enhanced ultrasonography; A2: longitudinal section of two-dimensional ultrasonography; A3: transverse section of contrast-enhanced ultrasonography; A4: transverse section of two-dimensional ultrasonography); C: postoperative ultrasonography at 1 months (C1: longitudinal section of contrast-enhanced ultrasonography; C2: longitudinal section of two-dimensional ultrasonography; C3: transverse section of contrast-enhanced ultrasonography; C4: transverse section of two-dimensional ultrasonography); D: postoperative ultrasonography at 3 months (D1: longitudinal section of contrast-enhanced ultrasonography; D2: longitudinal section of two-dimensional ultrasonography; D3: transverse section of contrast-enhanced ultrasonography; D4: transverse section of two-dimensional ultrasonography); E: postoperative ultrasonography at 6 months (E1: longitudinal section of contrast-enhanced ultrasonography; E2: longitudinal section of two-dimensional ultrasonography; E3: transverse section of contrast-enhanced ultrasonography; E4: transverse section of two-dimensional ultrasonography); F: postoperative ultrasonography at 12 months (F1: longitudinal section of contrast-enhanced ultrasonography; F2: longitudinal section of two-dimensional ultrasonography; F3: transverse section of contrast-enhanced ultrasonography; F4: transverse section of two-dimensional ultrasonography)

A total of 105 nodules in 105 patients were enrolled in the present study, with a mean age of 46.70 ± 13.05 years, and 87 of them (82.9%) were female. There were 49 solid nodules (46.7%), and 56 solid-cystic nodules (53.3%). The basic characteristics of the patients and nodules were summarized in Table 1. Preoperative and postoperative ultrasonography of a same patient were showed in Fig. 1. Voice change occurred in one patient (1.0%) and local hematoma occurred in two patients (1.9%), and all of them completely recovered at 3-month follow-up. No life-threatening complication and procedure-related death occurred perioperatively and during the follow-up.

**Table 1:** Characteristics of all patients and nodules

Variables	Characteristics
Age (years)^a^	46.70 ± 13.05
Gender, n (%)	
male	18 (17.1)
female	87 (82.9)
Nature, n (%)	
Solid	49 (46.7%)
Solid-cystic	56 (53.3%)
Vt (ml)^a^	13.38 ± 13.71
Vt < 10 ml	60
Vt 10-20 ml	25
Vt 20 ml -30 ml	11
Vt > 30 ml	9
Total energy	2.51 ± 1.84
Energy/volume	0.29 ± 0.18
Symptoms	
Compression and/or cosmic problem	67
Thyroid function tests^b^	
TSH	1.31 (0.85–2.06)
fT3	*4.74 (4.45–5.18)*
fT4	*15.79 (14.39–16.83)*

^a^mean ± standard deviation

^b^ median and interquartile range

### Dynamic change of nodule volumes

The median follow up time was 25.1 months (12 months to 36 months). During the follow up, regrowth occurred in 43 cases, suggested by a more than 50% increase of Vv in 39 cases, and a more than 50% increase of both Vv and Vt in 4 cases. The detailed volume information, as well as the number and time of regrowth were showed in Table 2.

**Table 2:** Nodule volume changes according to the follow-up period

Variables	0 month	1 month	3 month	6 month	12 month	24 month	36 month
No. of nodules	105	91	71	78	101	18	5
Vt^a^	8.65 (4.93–16.6)	3.74 (2.01–5.67)	2.41 (1.36–3.81)	1.79 (0.70–2.97)	1.09 (2.52–0.49)	0.84 (0.20–3.18)	0.37 (0.07–2.55)
Va^a^	8.38 (4.76–13.46)	2.39 (1.43–4.19)	1.31 (0.72–2.42)	0.73 (0.16–1.41)	0.28 (0.09–0.94)	0.23 (0.00–1.25)	0.03 (0.00–1.22)
Vv^a^	0.29(−1.07–4.00)	1.05 (0.38–2.39)	1.07 (0.36–2.17)	0.59 (0.21–1.52)	0.51 (0.14–1.65)	0.47 (0.11–1.94)	0.37 (0.05–1.33)
Vv increase ≥50%, n (%)	0	0	18 (25.4)	15 (19.2)	26 (25.7)	5 (2.8)	2 (0.4)
Vt increase ≥50%, n (%)	0	0	0	0	4 (4.0)	0	0

^a^median and interquartile range

### Dynamic change of nodules with regrowth

For the 43 nodules which underwent regrowth during follow-up, the accumulated rate of regrowth in different time points were calculated and summarized in Table 3. There was a significant difference between the rate of nodular regrowth on the 1st month, the 3rd month, the 6th month post-operatively and that on 36th month, while there was no significant between the rate of nodular regrowth on the 12th month, the 24th month postoperatively and that on the 36th month postoperatively. The rate of nodular regrowth on the 12th month postoperatively showed substantial consistency with that on the 36th month postoperatively (Kappa = 0.656).

**Table 3:** Accumulated cases showing regrowth in different follow-up time points

Time after RFA	Accumulated cases showing regrowth	Rate	P	Kappa
1 month	9	20.93%	0.000	0.013
3 month	20	46.51%	0.000	0.041
6 month	25	58.14%	0.000	0.064
12 month	41	95.35%	1.000	0.656
24 month	42	97.67%	1.000	1.000
36 month	43	100.00%	1.000	1.000

## Discussion

The dynamic change of volume of benign thyroid nodule after RFA were assessed using CEUS in the present study. The main results of this study showed that 95.35% of nodular regrowth occurred in 12 months after RFA, with no statistical significance compared to the total rate of nodular regrowth. Treatments were effective with low complication rate, and no significant complications like procedure-related deaths occurred during up to 3-year follow-up.

Marginal recurrence of ablated thyroid nodule had been firstly reported by Baek and his colleagues [9]. However, primary purpose of RFA for benign thyroid nodule is to conduct effective debulking or to reduce pressure symptoms, rather than to ablate the entire nodule [5], which is considered to be technically difficult, because complications might be inevitable due to the existence of critical structures around the thyroid, such as oesophagus, trachea, vagus nerve, recurrent laryngeal nerve [22], sympathetic ganglion and blood vessels [23]. However, incompletely treated nodule margin could be a reason of the regrowth of thyroid nodule [5]. Therefore, thyroid nodule should have their margin treated as much as possible to minimize marginal recurrence.

Previous studies had identified several factors influencing marginal regrowth of nodule. Initial solidity and nodule volume affects treatment response. Huh et al. had suggested that RFA is effective in most thyroid nodules, however, large nodules require more energy in order to achieve complete ablation of peripheries [10]. Ha et al. suggested a moving-shot technique to minimize marginal recurrence [24]. Internal cooled electrode could also minimize thermal damage to the surrounding critical structures of thyroid [1]. Thus, the current study applied moving-shot technique and internal cooled electrode. Moreover, other influencing factors of nodule regrowth includes nodule nature, maximum temperature reached during ablation treatment, treatment modalities, energy type and post-procedural marginal vascularity [25, 26].

Monitoring Vv increase is important to identify nodule regrowth and symptom recurrence. The change in Vt was considered to consist of a Va decrease and a Vv increase. In the earlier period after RFA, Vt becomes smaller when Va decrease is greater than Vv increase. In the later period after RFA, Vt becomes larger when Vv increase exceeds Va decrease (regrowth). Vv increase might be masked by Va decrease if Vt is the only measured value. Thus, measurement of Vv had been recommended to detect regrowth and to provide evidence for a secondary RFA [27].

Up to date, no widely-accepted consensus on the optimal timing of secondary RFA exist. Our study found that 95.35% of nodular regrowth occurred in 12 months after RFA, and the rate showed substantial consistency with that on the 36th month postoperatively (Kappa = 0.656). Thus, the 12th month after RFA might be the optimal time for volume assessment to make the decision of secondary RFAs. On the other hand, it should also be noted that at 6th month postoperatively, 58.14% of nodular regrowth had been detected, which means for these patients, evaluation at 6th month was also important. However, negative results at 6th month postoperatively could not be considered as the final result, because there is still a good chance to detect nodular regrowth at next time point.

In the present study, CEUS was used to evaluate the dynamic change of thyroid benign nodules after RFA, because previous studies have suggested that the ablation zone can be more clearly visualized with CEUS than with color Doppler US [28]. Dynamic changes of nodular volume were also evaluated using CEUS in several our previous studies.

We have to admitted that there were still several limitations of our study. First, the nature of the retrospective, small -sampled study made it impossible to avoid selection bias. Second, we did not compare results from non-contrast ultrasound and contrast ultrasound because lack of a gold-standard method as reference. Besides, changes of nodular volume could not be assessed after a > 50% increase of Vt or Vv was firstly found because lack of subsequent data. Moreover, complexity of dynamic change of the volume, different time-volume curve of different nodules, and lack of data after secondary RFAs, made the data imbalanced, which is why quantitative compare (for example analysis of variance) could not be used. The interval of follow-up time points might also be inappropriate, lacking data after 9 months or 15 months, due to the retrospective nature of the study, as well as the cost-effective need in clinical routine. A further large volume, real world study with specifically designed follow-up time points might be more convincing in assessing the dynamic change of nodular volume after RFA. However, based on the existed results, we are still confident to conclude that the majority cases of regrowth occurred in 12 months after RFA.

## Conclusions

CEUS was an effective and safe tool to monitor volume change of benign thyroid nodules after RFA. The majority cases of regrowth occurred in 12 months after RFA, thus, the 12th month after RFA might be the optimal time for volume assessment to make the decision of secondary RFAs.

## Data Availability

In attempt to preserve the privacy of participants, data of participants will not be shared; data can be available from authors upon request.

## Abbreviations

CEUS: Contrast-enhanced ultrasonography
RFA: Radiofrequency ablation
Va: Ablated volume
Vv: Vital volume
Vt: Total volume
